# A balanced approach for stable hips in children with cerebral palsy: a combination of moderate VDRO and pelvic osteotomy

**DOI:** 10.1007/s11832-016-0753-5

**Published:** 2016-06-27

**Authors:** Kerstin Reidy, Christoph Heidt, Stefan Dierauer, Hanspeter Huber

**Affiliations:** Department of Orthopaedic Surgery, University Children’s Hospital Zurich, Steinwiesstrasse 75, 8032 Zurich, Switzerland; Department of Orthopaedic Surgery, Cantonal Hospital Winterthur, Brauerstrasse 15, 8401 Winterthur, Switzerland

**Keywords:** Cerebral palsy, VDRO, Hip dysplasia, Hip reconstruction, Pelvic osteotomy, Neck shaft angle

## Abstract

**Background:**

Hip reconstructive surgery in cerebral palsy (CP) patients necessitates either femoral varus derotational osteotomy (VDRO) or pelvic osteotomy, or both. The purpose of this study is to review the results of a moderate varisation [planned neck shaft angle (NSA) of 130°] in combination with pelvic osteotomy for a consecutive series of patients.

**Methods:**

Patients with CP who had been treated at our institution for hip dysplasia, subluxation or dislocation with VDRO in combination with pelvic osteotomy between 2005 and 2010 were reviewed.

**Results:**

Forty patients with a mean follow-up of 5.4 years were included. The mean age at the time of operation was 8.9 years. The majority were non-ambulant children [GMFCS I–III: *n* = 11 (27.5 %); GMFCS IV–V: *n* = 29 (72.5 %)]. In total, 57 hips were treated with both femoral and pelvic osteotomy. The mean pre-operative NSA angle of 152.3° was reduced to 132.6° post-operatively. Additional adductor tenotomy was performed in nine hips (16 %) at initial operation. Reimers’ migration percentage (MP) was improved from 63.6 % pre-operatively to 2.7 % post-operatively and showed a mean of 9.7 % at the final review. The results were good in 96.5 % (*n* = 55) with centred, stable hips (MP <33 %), fair in one with a subluxated hip (MP 42 %) and poor in one requiring revision pelvic osteotomy for ventral instability.

**Conclusions:**

This approach maintains good hip abduction and reduces soft-tissue surgery. Moderate varisation in VDRO in combination with pelvic osteotomy leads to good mid-term results with stable, pain-free hips, even in patients with severe spastic quadriplegia.

## Introduction

Spastic hip subluxation or dislocation is a frequent problem in children with cerebral palsy (CP). It is present among all functional levels but its frequency correlates clearly with the severity of the disease. Only 7 % of independent ambulators but up to 60–90 % of non-walking patients with severe spastic quadriplegia are affected [1].

Possible clinical problems associated with spasticity and hip instability include restriction of the ability to stand and walk, sitting imbalance, diminished hip motion, prevention of further motor progression, difficulty with perineal care and pain [1–3]. Unilateral dislocation may cause pelvic obliquity and attribute to scoliosis [4, 5].

For the treating surgeon, several surgical procedures are available to restore hip stability. Most commonly, combinations of soft-tissue balancing measures, femoral varus derotational osteotomy (VDRO) and pelvic osteotomies are used. It is known that the effect of the corrective femoral osteotomy alone on hip centration may not be sufficient, especially in severely subluxated or dislocated joints in patients with higher GMFCS levels [6]. The overall results are better when all existing deformities of the pelvis and the femur are corrected [2, 3, 7–12]. To achieve this goal, multiple surgical procedures are often performed. However, there remains controversy about the amount of surgery that is required to stabilise the hip [13, 14]. Even with multiple surgical procedures, re-dislocations must be expected in 6–7 % of cases [3].

It has been shown that the neck shaft angle (NSA) increases over time after a VDRO. Therefore, some authors suggest that children with higher GMFCS levels may require a higher amount of varisation with extensive soft-tissue release to maintain hip stability, accepting the possible higher risk of avascular necrosis (AVN) [15, 16]. Regarding the amount of VDRO, no consensus exists on how much correction of the proximal femur should be executed. One of the rare recommendations by Miller et al. [15] suggests a correction to a post-operative NSA down to 100° in non-walking patients and 120° in walking patients, mostly in conjunction with an extensive release of the adductors.

At our centre, we perform a combination of a pelvic osteotomy and a moderate VDRO with a planned post-operative NSA of 130° in a similar manner across all GMFCS levels. We believe that this will avoid a release of the adductors in most cases and still lead to stable, pain-free hips. The purpose of this paper is to review our results, to evaluate the frequency of complications, the necessity of soft-tissue releases and to report the radiologic and clinical outcome regarding hip stability.

## Patients and methods

Following approval from the ethical committee, we retrospectively reviewed the X-rays and data sheets of all paediatric patients with CP who had been treated in our institution between 2005 and 2010 for hip dysplasia, subluxation or dislocation with a combined pelvic osteotomy and VDRO. Included were all patients who had a minimum follow-up of 2 years clinically and radiographically. Patients with prior bony or soft-tissue operations at the level of the hip were excluded, as well as patients with neurological disorders other than infantile CP. Antispasmodic therapeutics such as baclofen, phenol or botox injections were not taken into consideration.

### Technical description

**Fig. 1:**
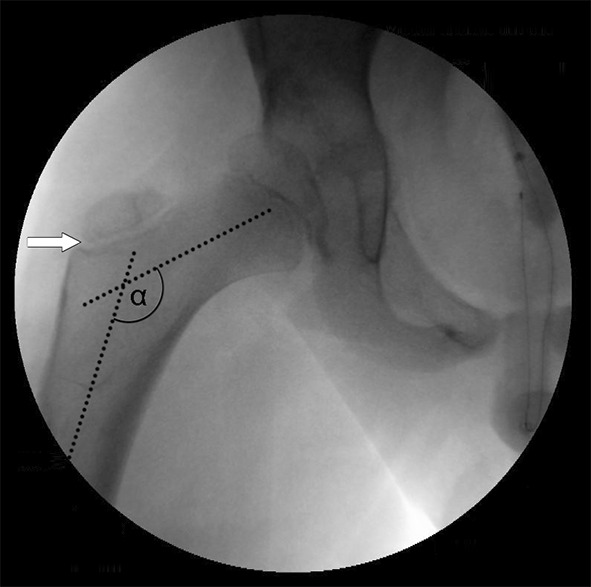
Example of intra-operative measurement of the neck shaft angle (NSA). For correct measurements and corrected anteversion, the growth plate of the greater trochanter must be fully visible (*white arrow*). The pre-operative NSA was measured on an image intensifier and correction was adjusted to the measured value

Prior to surgical intervention, computed tomography (CT) scans with 3D reconstructions were performed to visualise the direction of migration, as well as the sphericity and the bony deformity. For the pelvic osteotomy, a Smith–Peterson approach was used. The type of osteotomy was adjusted to the predominant direction of instability: Dega osteotomy for a more posterior, Pemberton osteotomy for a more anterior instability or for a multi-directional instability. In all cases, VDRO was performed through a subvastus approach. It included a varisation, derotation and shortening. The amount of correction was adjusted to the individual situation: intra-operatively correct rotated a.p. X-rays of the proximal femur were obtained and the amount of femoral varisation was defined with a goal of an NSA of around 130° after varisation (Fig. 1). The amount of femoral shortening depended on the height of bone graft needed for inter-position on the pelvic osteotomy side. An additional 5 mm was taken to reduce tension for an easier and more gentle reduction of the hip joint. By reducing pressure on the hip joint and, thereby, on the femoral head, the risk of AVN can be decreased. Fixation was achieved with either an AO blade plate or as described by Rutz and Brunner with an LCP paediatric hip plate (Synthes, Grenchen, Switzerland) [17]. In all cases, an intra-pelvine lengthening of the iliopsoas tendon was performed. An additional adductor tenotomy was only done if intra-operative hip abduction was less than 20° after pelvic osteotomy and VDRO. If necessary, an abductor release was performed in the case of a fixed abduction contracture, mostly after reduction of an anterior hip dislocation. Post-operatively, hip spica cast was applied for at least 2 weeks, primarily for analgetic reasons, in some cases prolonged for 4–6 weeks, depending on bone quality and activity level. Walking patients older than 10 years at operation were not immobilised in a hip spica cast post-operatively.

### Analysis

Gender, GMFCS level, age at surgery and details of the operation were recorded in a spreadsheet. In patients with bilateral surgery, each hip was reviewed independently, except for age analysis. Pre-, post-operative and the latest follow-up radiographs were analysed for NSA as well as migration percentage (MP) by a consultant orthopaedic surgeon (KR). According to Reimers, a hip was regarded as centred with an MP <33 %, subluxated with an MP of at least 33 % and dislocated with an MP of 100 % [18]. With regards to this, we classified the hips as good (MP <33 %), fair (MP between 33 and 100 %) and poor (MP 100 %). Further, complications were recorded and the presence of osteonecrosis was evaluated.

### Statistical methods

All analyses were performed using SPSS 22.0 (Armonk, NY, USA). Geometric means and relative standard deviations were calculated for continuous variables. The Mann–Whitney *U* was used for baseline measurements. One-way analysis of variance (ANOVA) with post hoc testing was used to examine differences in the NSA and MP pre-, post-operative and last follow-up measurements (level of significance 0.05). Cross-tabulation and the Chi-square test was used for the analysis of adductor release surgery among the two groups (walkers and non-walkers).

## Results

Eighty-three paediatric patients were treated operatively for neurologic hip dysplasia at our institution between 2005 and 2010. Out of 62 patients with cerebral palsy, 22 did not meet our inclusion criteria.

Forty patients (20 males and 20 females) were included in our study. Twenty-nine patients (72.5 %) were non-walkers (GMFCS IV or V) and 11 patients (27.5 %) were walkers (GMFCS I, II or III) (Table 1). The average patient age at surgery was 8.9 years (range 2.6–14.8 years), 9.3 years among walkers (range 2.6–13.3 years) and 8.8 years among non-walkers (range 4.3–14.7). The average duration of follow-up was 65.4 months (range 24–123 months). The average age at final follow-up was 14.4 years (range 9.1–20.4 years). Mann–Whitney *U* testing did not show any difference between groups (walkers/non-walkers) in regards to baseline measurements.

**Table 1:** Patient distribution across GMFCS levels

GMFCS	Patients		Hips	
	*n*	%	*n*	%
Walkers	11	27.5	13	22.8
I	2		2	
II	5		6	
III	4		5	
Non-walkers	29	72.5	44	77.2
IV	4		5	
V	25		39	

According to Reimers, nine hips were centred, 35 subluxated and 13 dislocated at the time of operation. For all nine centred hips (nine patients), dysplastic acetabular roof changes were documented. Six of these nine patients were walkers. The other three centred hips were operated in conjunction with a combined procedure of the contralateral hip which showed a subluxation or dislocation. In total, 57 pelvic osteotomies in combination with VDRO were performed. Seventeen patients (42.5 %) were treated with a bilateral pelvic and femoral osteotomy, and 23 patients (57.5 %) had a combined pelvic osteotomy and VDRO only on one side. Thirty-four Dega osteotomies were performed in hips with a more posterior instability. In four hips with a planned Dega osteotomy for posterior instability, the inner table of the ilium was perforated while levering down the acetabular roof, resulting in a more Pemberton type osteotomy. Altogether, 19 Pemberton osteotomies were performed: seven hips showed an isolated anterior and 12 hips a multi-directional instability. The mean pre-operative NSA was 152.3° (range 142°–160°), as measured in standardised positioning on fluoroscopy intra-operatively. The average planned varisation was 16.9° (range 10°–30°), resulting in an average planned post-operative NSA of 135.3° (range 126°–147°). An average external rotation of 20.9° (range 5°–30°) and shortening of 18.0 mm (range 0–35 mm) was executed. For osteotomy fixation, an AO blade plate was used in 38 hips (66.7 %) and an LCP paediatric hip plate in 19 hips (33.3 %). At the time of index surgery, additional soft-tissue release was performed in 13 hips (22.8 %): nine adductor tenotomies and in four hips, a release of the abductors (two patients with bilateral anterior hip dislocation).

**Fig. 2:**
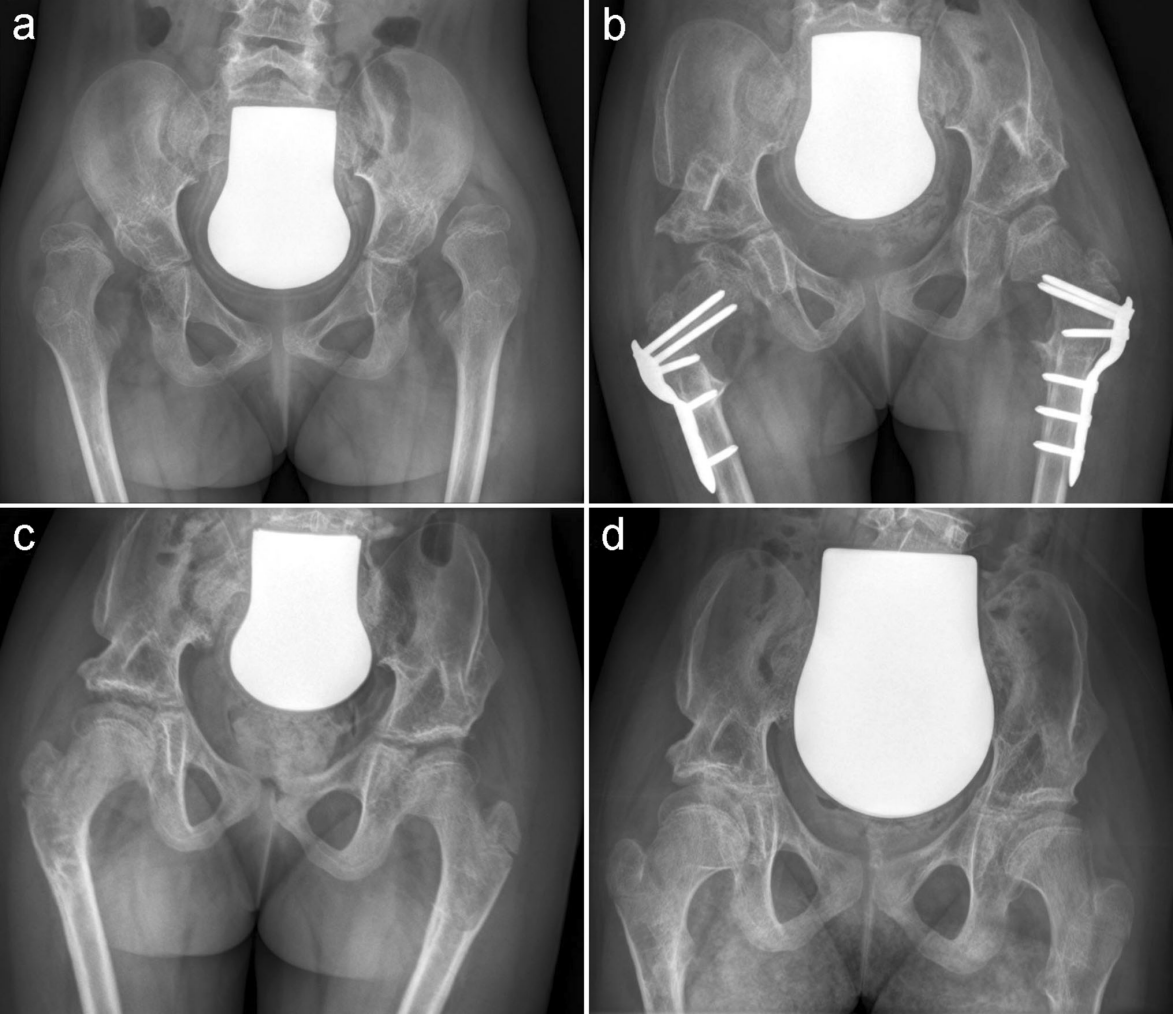
Female, aged 7.2 years, GMFCS V. **a** Pre-operative: right hip MP 100 %, NSA 145°, left hip MP 100 %, NSA 145°. **b** Post-operative NSA: right hip 128°, left hip 132°. **c** Two years post-operative. **d** Five years post-operative: right hip MP 16 %, NSA 131°, left hip MP 21 %, NSA 137°

The mean measured post-operative NSA was 132.6° (range 115°–146°). At the latest follow-up, the mean NSA was 137.2° (range 116°–159°) (Fig. 2). Pre-operatively, we found in non-walkers a slightly higher NSA than in walkers (not significant). The post-operative NSA in non-walkers was significantly lower than in walkers (131.5° vs. 136.3°, *p* < 0.011). Interestingly, non-walkers showed less elevation of the NSA (131.5°–135.4°) compared to walkers (136.3°–143.8°) at final follow-up (Table 2). Reimers’ MP could be improved from a mean of 63.6 % pre-operatively to 2.7 % post-operatively. At the latest follow-up, the mean MP was 9.7 % (Table 2). We did not see any statistical difference between walkers and non-walkers regarding the MP.

**Table 2:** Pre- and post-operative radiographic measurements

	All	Walkers (*n* = 13, 22 %)	Non-walkers (*n* = 46, 78 %)
NSA (°)			
Pre-operative	152.3 ± 6.7	150.3 ± 4.5	152.8 ± 7.1
Correction	19.7 ± 9.3	14.0 ± 6.7	21.3 ± 9.4*^1^
Post-operative	132.6 ± 9.7	136.3 ± 7.5*	131.5 ± 10.1*
Last follow-up	137.2 ± 10.1	143.8 ± 11.0***	135.4 ± 9.1**
MP (%)			
Pre-operative	63.6 ± 26.9	47.7 ± 22.4	68.3 ± 26.6
Post-operative	2.7 ± 5.9	8.2 ± 8.9*	1.2 ± 3.5*
Last follow-up	9.7 ± 9.2	12.2 ± 6.7*	8.9 ± 9.7*

Values ± standard deviation

*p*-Values account for inter-group to pre-operative comparison if not marked separately

*p*-Values: * *p* < 0.0001, *^1^ < 0.011 (inter-group comparison), ** *p* = 0.049, *** NSS (not statistically significant)

During follow-up, another soft-tissue release was performed in seven hips between 6 and 57 months after the initial procedure: four hips had a tenotomy of the adductors and an abductor release was performed in three hips. Three of these seven hips already had a soft-tissue release during the first surgery. In total, 17 out of 57 hips (29.8 %) had a soft-tissue release other than iliopsoas lengthening during the initial procedure and/or the post-operative course. We did not find a statistical difference in the total amount of adductor releases in walkers and non-walkers. The final results were good, with stable, centred hips (MP <33 %) in 96.5 % of the hips (*n* = 55). A fair result was recorded in one hip with a recurrent subluxation (MP 42 %). One hip was classified as poor despite an MP of 0 % at the latest follow-up because of a necessary revision due to painful anterior hip instability 9 months after initial operation. Two patients needed early operative revisions: in one case, a re-osteosynthesis had to be performed for varus malunion due to implant failure in poor bone quality. One patient suffered from post-operative soft-tissue infection that needed debridement and antibiotic therapy.

The outpatient records revealed that none of the patients suffered from hip pain at the latest follow-up.

## Discussion

The concept of hip reconstruction in children with CP is a widely accepted treatment for spastic hip sub- and dislocation. Although an effective treatment, the combination of femoral varus derotation and pelvic osteotomy was found to have a complication rate of up to 30 % [13] and recurrence rates of sub- and dislocated hips are reported in 5–10 % at long-term follow-up [3, 19–21].

According to Rutz et al. [22], pre-operative MP was the most influential risk factor affecting the post-operative outcome, suggesting hip reconstruction at an early stage. In our study, 35 subluxated and 13 dislocated hips were treated with hip reconstruction, having a low complication rate concerning re-dislocation. We also agree to, instead, perform a hip reconstruction including both femoral varus derotation and pelvic osteotomy at an early stage to reduce the re-dislocation rate.

A review of the current literature shows that there is no consensus or clear recommendation on how much the NSA should be corrected in varus to achieve and maintain stable hips with the lowest complication rates (Table 3). One of the rare recommendations suggests a correction to the post-operative NSA to 100° in non-walking patients and 120° in walking patients, mostly in conjunction with an extensive release of the adductors [15]. Rutz et al. aimed at a post-operative NSA of 120–125°, with recurrent hip dislocations in 2 out of 168 hips. The extent of soft-tissue releases was not recorded [22]. In our study, we combined moderate varisation with a post-operative NSA of 132.6° with restrained release of the adductors and had a low complication rate concerning instability, in walkers and non-walkers as well. Recently, Bayusentono and colleagues [23] recommended adequate varisation at the time of the primary surgery, although they did not find significant loss of correction of the NSA when analysing more than 800 radiographs over a period of 12 years. They found a statistically significant increase of the MP of 2.0 % per year in GMFCS level IV and 3.5 % in level V, which corresponds to our increase of 4.0 % in walkers and 7.7 % in non-walkers during a mean follow-up of more than 5 years. Jóźwiak et al. [16] indicate, in another long-term analysis, that the loss of correction is progressive. Therefore, the authors stated to use a more extensive soft-tissue release and a more radical VDRO.

**Table 3:** Literature review

	Hips (patients)	Mean age (years)	NSA (°) pre-op			NSA (°) post-op	NSA (°) last FU	FU (years)	± Soft-tissue		Outcomes (definition)	Complications	Comment
Mubarak et al. [10]	18 (11)	8.5	149			NR	95	6.8	Release of adductors, psoas and proximal hamstrings in all		17 <30 % MP (all pain-free hips)	2 AVN, 1 dislocation, 1 fracture	Pre-op 17 with MP >50 %
Miller et al. [15]	59 (51)	9.9	150 (148)			104 (105)	115 (114)	2.7	49 (83 %) adductor lengthening		Pain-free hips 82 % (14/18)	2 Re-dislocations requiring surgery	
Debnath et al. [29]	12 (11)	14.1	147.2			NR	128.6	13.1	Psoas lengthening and adductor release in all		11 Good, 1 fair, MP post-op 13.7 %	2 OA; 1 HO, 1 fracture	
Al-Ghadir et al. [25]	39 (36)	9.4	153.4			NR	120.8	4.1	Release of adductors, psoas and proximal hamstrings in all		MP 10.4 % post-op	None	All pain-free
Khouri et al. [26]	59 (52)	7.4	145			120	125	>1			MP 25 % post-op	No major complication	
Huh et al. [27]	24 (75)	7	162			128	NR	4.6	Open reduction in all		MP 28 % post-op	1 Revision, 2 minor complications (infection, ulcer, prolonged pain)	
Davids et al. [30]	137 (75)	7	151			105	119	5.5	NR		MI 1.41	5 Delayed unions, 6 AVN, 4 dislocations, 1 fracture	
Dhawale et al. [28]	22 (19)	7.5	150.7			112	120	11.7	17 (77 %) adductor releases			21 % complications, 1 dislocation, 1 subluxation, 1 coxa vara + add. deformity, 1 fracture, 1 infection	
McNerney et al. [19]	104 (75)	8.1	NR			NR	NR	6.9	NR		MP 5 % post-op MP 14 % FU	44 Complications, 1 extension of acetabuloplasty into hip joint, 8 AVN, 2 coxa vara, 1 windblown hip, 3 wound infections, 7 HO, 32 re-surgeries to maintain hip stability	NSA 110°–120° desired
Rutz et al. [22]	121 (168)	11.3	NR			NR	NR	7.3	NR			Surgical complication rate 10.5 %	NSA 120°–125° desired
Mallet [21]	20 (20)	8.1	153			115	130	9.1	Adductor longus release in all		MP 14.6 % post-op	1 Dislocation, 5 subluxations, 1 septic pseudarthrosis	

*AI* acetabular index, *CEA* centre-edge angle, *FU* follow-up, *MP* migration percentage, *OA* osteoarthritis, *SAA* Sharp’s acetabular angle, *NR* not reported, *HO* heterotopic ossifications, *MI* medialisation index

According to the literature, even in combined VDRO and pelvic osteotomy, releases of the adductors are carried out in up to 80 % of patients [15, 23–27]. It is known that soft-tissue corrections have a less predictable outcome than bony procedures, e.g. an extensive adductor release may fail due to scarring. We, therefore, only perform adductor release if abduction does not reach at least 20° at the end of surgery when the shortening of the femur has already lead to some relative lengthening of the muscles, even though our results are comparable to other series, in which a greater amount of varisation was performed and adductor release was carried out in 70–100 % [15, 21, 28, 29]. Miller et al., for example, performed an adductor release at the beginning of the surgery if the abduction did not exceed 45° [15] and McNerney et al. performed adductor lengthening to obtain abduction of 60° [19].

One of the possible complications of proximal femoral varisation is AVN of the femoral head. There has been discussion as to whether excessive varisation may lead to AVN [24]. A study comparing two different techniques for femoral varisation (end-to-side and end-to-end), which resulted in a post-operative angle of around 100° in both cases, reported an 5.5–6 % AVN rate [30]. We did not find any signs of AVN in our collective during the whole radiologic follow-up.

For 96.5 % of stable hips with an MP <33 % after an average of 5.4 years, our results are comparable to other studies (Table 3). Our mid-term results were able to show that even a moderate VDRO in combination with a pelvic osteotomy is a safe way to generate stable, pain-free hips. Outcomes were good in 96.5 % of patients and we performed adductor releases in only 13 hips (22.8 %).

There are important drawbacks to our analysis. Foremost, only around 50 % of patients reached skeletal maturity at the time of the latest follow-up. Our mean follow-up was 5.4 years; therefore, long-term results need to verify the effectiveness of this treatment. Secondly, the valgus position of the proximal femur is often overestimated on a.p. radiographs due to oblique projection caused by high femoral anteversion. The measurements of the NSA were only taken on a standardised and correctly rotated a.p. view according to Laplaza and Root intra-operatively [31]. X-rays taken post-operatively and during the latest follow-up often showed some rotation, which has a well-known impact on the measurement of the NSA. The measured NSA will, rather, be higher on a not correctly rotated X-ray and the loss of correction of the NSA at last follow-up (4.6° on average in our study) is rather over- than underestimated. Routinely, radiographic follow-up included pelvic a.p. X-rays only. In case of clinical suspicion for an anterior progressive subluxation, we do indeed perform a CT scan with 3D reconstruction. Still, anterior progressive subluxation might be missed or not be detected at an early stage.

We were able to show that our approach of a moderate VDRO in combination with a pelvic osteotomy has good mid-term results with stable and pain-free hips. Using this technique even in patients with severe spastic quadriplegia is safe and the amount of soft-tissue surgery can be limited.
